# Distinguishing the rates of gene activation from phenotypic variations

**DOI:** 10.1186/s12918-015-0172-0

**Published:** 2015-06-18

**Authors:** Ye Chen, Cheng Lv, Fangting Li, Tiejun Li

**Affiliations:** LMAM and School of Mathematical Sciences, Peking University, No. 5 Yiheyuan Road, Beijing, 100871 China; School of Physics, Peking University, No. 5 Yiheyuan Road, Beijing, 100871 China; Center for Quantitative Biology, Peking University, No. 5 Yiheyuan Road, Beijing, 100871 China

**Keywords:** Gene expression, Positive feedback, Energy landscape, Flow cytometry experiment

## Abstract

**Background:**

Stochastic genetic switching driven by intrinsic noise is an important process in gene expression. When the rates of gene activation/inactivation are relatively slow, fast, or medium compared with the synthesis/degradation rates of mRNAs and proteins, the variability of protein and mRNA levels may exhibit very different dynamical patterns. It is desirable to provide a systematic approach to identify their key dynamical features in different regimes, aiming at distinguishing which regime a considered gene regulatory network is in from their phenotypic variations.

**Results:**

We studied a gene expression model with positive feedbacks when genetic switching rates vary over a wide range. With the goal of providing a method to distinguish the regime of the switching rates, we first focus on understanding the essential dynamics of gene expression system in different cases. In the regime of slow switching rates, we found that the effective dynamics can be reduced to independent evolutions on two separate layers corresponding to gene activation and inactivation states, and the transitions between two layers are rare events, after which the system goes mainly along deterministic ODE trajectories on a particular layer to reach new steady states. The energy landscape in this regime can be well approximated by using Gaussian mixture model. In the regime of intermediate switching rates, we analyzed the mean switching time to investigate the stability of the system in different parameter ranges. We also discussed the case of fast switching rates from the viewpoint of transition state theory. Based on the obtained results, we made a proposal to distinguish these three regimes in a simulation experiment. We identified the intermediate regime from the fact that the strength of cellular memory is lower than the other two cases, and the fast and slow regimes can be distinguished by their different perturbation-response behavior with respect to the switching rates perturbations.

**Conclusions:**

We proposed a simulation experiment to distinguish the slow, intermediate and fast regimes, which is the main point of our paper. In order to achieve this goal, we systematically studied the essential dynamics of gene expression system when the switching rates are in different regimes. Our theoretical understanding provides new insights on the gene expression experiments.

**Electronic supplementary material:**

The online version of this article (doi:10.1186/s12918-015-0172-0) contains supplementary material, which is available to authorized users.

## Background

Stochasticity is an inherent property of living cells. Stochastic perturbation comes not only from the noisy environment that cells live in, but also from all processes going on inside cells [1]. Especially when the copy number of molecules like mRNA or protein gets smaller, stochastic fluctuations can have significant effect on the behavior of a cell. There have been many studies focused on discovering the effect of noise in living systems [2, 3], and some of the results show that cells do not only evolve to resist noise perturbation better, but also form massive mechanisms which are the benefits of stochasticity [4, 5]. Genetic switching is a typical example of noise-driven cellular behavior which can be performed spontaneously under particular conditions in living cells, such as the switch of *lac* operon in Escherichia coli [6], the formation of biofilm [7], and the entrance into competence state [8] in Bacillus subtilis, etc.

Several recent studies discovered strong connections between noise and gene regulation mechanisms through measuring variability in protein and mRNA levels [9, 10]. Both experimental and theoretical results suggested that the switching rates of gene between its active and inactive states have significant effects on the noise of gene expression [11, 12] and are highly responsible to different gene expression dynamics. Previous theoretical studies mainly focused on the regime with fast switching rates [13–15], however, recently some unique phenomena are observed especially when switching rates are slow, either due to the chromatin remodeling precess [16] or the transportation of transcription factor into nucleus [17]. Thus, it is important and desirable to provide an approach which can effectively identify the key dynamical features of gene expression system when switching rates are slow, and to distinguish the regime of gene regulatory networks from different phenotypic variations.

In this paper, we aim at providing a method to distinguish the rates of genetic switches from phenotypic variations. First, we systematically investigated the dynamical and stochastic properties of different genetic regulatory models in a comparative way when biochemical rates for gene activation/inactivation, transcription, translation, and mRNA/protein degradation vary over a wide range. We focused on a two-state gene-expression model with positive feedback loop, which is simplified from a four-state gene-expression network taking account of chromatin opening/closing step and regulatory protein binding/unbinding step [16, 18]. In the regime when the rates of gene activation/inactivation are relatively small compared with the synthesis/degradation rates of mRNAs and proteins, we found that the effective dynamics can be reduced to independent evolutions on two separate layers. We reconstructed the energy landscape of the system through Gaussian mixture approximations, and it is qualitatively different from the previously known picture in [15, 19]. In the regime with relatively fast switching rates, we briefly discussed about the system based on our previous study with large deviation theory [15]. In the regime with intermediate switching rates, we utilized the mean switching time (MST) to investigate the stability of the system in different parameter ranges. In the following paragraphs, we will call these three regimes as slow, fast, and intermediate regimes for convenience. Finally, based on our theoretical understanding of the essential dynamics of the slow, intermediate and fast regimes, we designed a simulation experiment to distinguish the three regimes. We identified the intermediate regime from the fact that cellular memory is much weaker in this regime than the other two regimes. And we distinguished the slow and fast regimes through their different response behavior with respect to switching rates perturbations. Our study gives a global characterization of the genetic switching dynamics in various cells and has potential guidance in real experiments.

## Methods

**Fig. 1:**
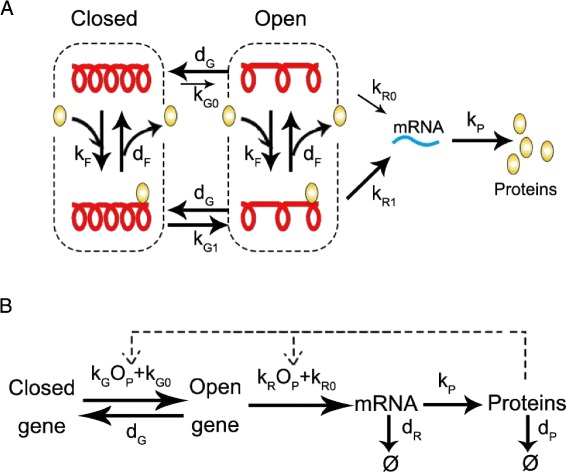
Schematic representation of the genetic switching model with positive feedback. **a** Four-state model. A single regulatory protein can bound to the DNA and improve the efficiency of chromatin opening. When the chromatin is open, mRNA is synthesized and will later be translated into a regulatory protein. The four states of DNA are (i) closed and unbounded, (ii) closed and bounded, (iii) open and unbounded, (iv) open and bounded. The switches between the adjacent states are reversible. **b** Simplified two-state model. Binding/unbinding is reduced through the QSSA

We consider a four-state gene-expression model with positive feedback loop (Fig. 1a). Proteins are self-activators in this system, since they can bind to DNA and activate the transcription step. Chromatin opening becomes relatively easy when DNA is bounded by a monomer regulatory protein. Open chromatin structure is permissible for transcription whereas closed chromatin is not. The rate of mRNA production reaches its maximum on the bounded and open state of DNA. Additionally, proteins and mRNAs are subject to degradation, which is not shown in Fig. 1a. Assuming that binding and release of regulatory proteins are in fast equilibrium [20, 21], this four-state model can be simplified into a two-state model (Fig. 1b) through the quasi-steady-state approximation (QSSA) (see Additional file 1 for derivation details) [22, 23]. We will say that the gene is *open* (*closed*) when the chromatin is open (closed), and the system switches between open and closed gene states through gene activation/inactivation steps.

In the two-state model, there are six reactions occurring among four chemical species. All of the reactions involved in this system are summarized in Table 1. Here *m* and *n* are the numbers of protein molecules and mRNA molecules, respectively. The jump rate into the open state and the transcription rate are controlled by the relative occupancy of protein at DNA binding site (i.e. *O*_*P*_), whereas the jump rate into the closed state is not affected by the number of proteins. *O*_*P*_ has the form *O*_*P*_=*n*/(*n*+*K*), where *K* is a constant by reduction and represents the strength of positive feedback. It is actually the conditional probability of state (iv) in Fig. 1a given that the chromatin is open. The rates *k*_*G*_*O*_*P*_+*k*_*G*0_ and *k*_*R*_*O*_*P*_+*k*_*R*0_ characterize the gene activation and mRNA synthesis, which are dependent of the number of proteins. The constants *d*_*G*_, *k*_*P*_, *d*_*R*_ and *d*_*P*_ correspond to the rates of gene inactivation, protein synthesis, mRNA and protein degradation. In fact, if we assume that dimer proteins (or other multimer-type of proteins) regulate the gene instead of the monomer protein, *O*_*P*_ will have the form *O*_*P*_=*n*^*k*^/(*n*^*k*^+*K*^*k*^). The value of *k* will not influence the essential dynamics of the gene expression model, on condition that we choose proper range of parameters with respect to different *k*. Under some reasonable assumptions, most of the derivations and results in the next section are still correct (see Additional file 1 for details).

**Table 1:** Reactions in the two-state gene expression network with positive feedback

Number		Reaction		
1	DNA _open_	$\xrightarrow {\qquad d_{G} \qquad }$	DNA _closed_	
2	DNA _closed_	$\xrightarrow {\;\;\;\;k_{G}O_{P}+K_{G0}\;\;\;\;}$	DNA _open_	
3	DNA _open_	$\xrightarrow {\;\;\;\;k_{R}O_{P}+K_{R0}\;\;\;\;}$	DNA _open_+*m*	
4	*m*	$\xrightarrow {\qquad k_{P}\qquad }$	*n*	
5	*m*	$\xrightarrow {\qquad d_{R}\qquad }$	*∅*	
6	*n*	$\xrightarrow {\qquad d_{P}\qquad }$	*∅*	

Due to the intrinsic noise, the dynamics of the system can be described by the chemical master equation (CME) [1, 24–27] as

(1) $${} \begin{aligned} \frac{\mathrm{d}P_{0}(m,n)}{\mathrm{d}t}&=-\left(k_{G0}+k_{G}\frac{n}{n+K}\right)P_{0}(m,n)\\ & \quad +d_{G}P_{1}(m,n)+k_{P}[P_{0}(m,n-1)-P_{0}(m,n)]\\ & \quad +d_{R}[(m+1)P_{0}(m+1,n)-mP_{0}(m,n)]\\ & \quad +d_{P}[(n+1)P_{0}(m,n+1)-nP_{0}(m,n)], \end{aligned}  $$

(2) $${} \begin{aligned} \frac{\mathrm{d}P_{1}(m,n)}{\mathrm{d}t}&=\left(k_{G0}+k_{G}\frac{n}{n+K}\right)P_{0}(m,n)\\ & \quad -d_{G}P_{1}(m,n)+k_{P}[P_{1}(m,n-1)-P_{1}(m,n)]\\ & \quad +\left(\!k_{R0}+k_{R}\frac{n}{n+K}\!\!\right)\!\left[P_{1}(m-1,n)\!-P_{1}(m,n)\right]\\ & \quad +d_{R}[(m+1)P_{1}(m+1,n)-mP_{1}(m,n)]\\ & \quad +d_{P}[(n+1)P_{1}(m,n+1)-nP_{1}(m,n)], \end{aligned}  $$

where *P*_*α*_(*m*,*n*) stands for the probability that there are *m* mRNA molecules and *n* protein molecules in the system when the gene is open (*α*=1) or closed (*α*=0). One possibility to study the behavior of the system is to numerically solve Eqs. 1-(2) by truncating the domain and setting the boundary conditions *P*_*α*_(*m*,*n*)=0 when $m\geqslant M$, $n\geqslant N$ (*M*,*N* is large enough, *α*=0,1). But simply obtaining this solution does not mean we understand the essential mechanism of the dynamics, and the computational effort may be huge when the mean number of molecules is quite large in this system. Thus, we aim to find other ways to learn about the dynamics.

We define a key parameter *κ* to compare two characteristic timescales of the problem: the timescale $d_{P}^{-1}$ on which the gene state changes and the timescale $d_{G}^{-1}$ on which the number of protein and mRNA molecules changes, where $d_{P}^{-1}$ is the lifetime of a single protein and $d_{G}^{-1}$ is the lifetime of the open gene state. The ratio *κ*=*d*_*G*_/*d*_*P*_ can take any positive value, which will lead to notable differences in the dynamics. Small ratios (*κ*≪0.01) describe long timescales of the change in gene states, compared with the timescale on which mRNA and protein numbers change. In this regime, there is enough time for the system to reach a steady state when gene is either open or closed. Transition from one steady state to another always occurs immediately after the genetic switches. Large values of the ratio (*κ*≫1) indicate that the gene states change very rapidly, whereas the synthesis and degradation of protein and mRNA molecules are relatively slow. As *κ* goes to infinity, the system exhibits the deterministic features [13, 15]. When *κ* is neither large nor small enough, genetic switches, transcription and translation will take place on the same timescale. The dynamics is complicate in this regime, and analytical results are hard to reach. So we resort to numerical simulations and extract useful information. In all of the three regimes, metastable states may occur with proper choice of parameters. Indeed, we will utilize a set of biologically relevant parameters in later computations (see Additional file 1 for details). For convenience, we will call the steady state with relatively large number of proteins as *on* state, whereas the steady state with relatively small number of proteins is *off* state.

## Results

### Case A: slow regime

**Fig. 2:**
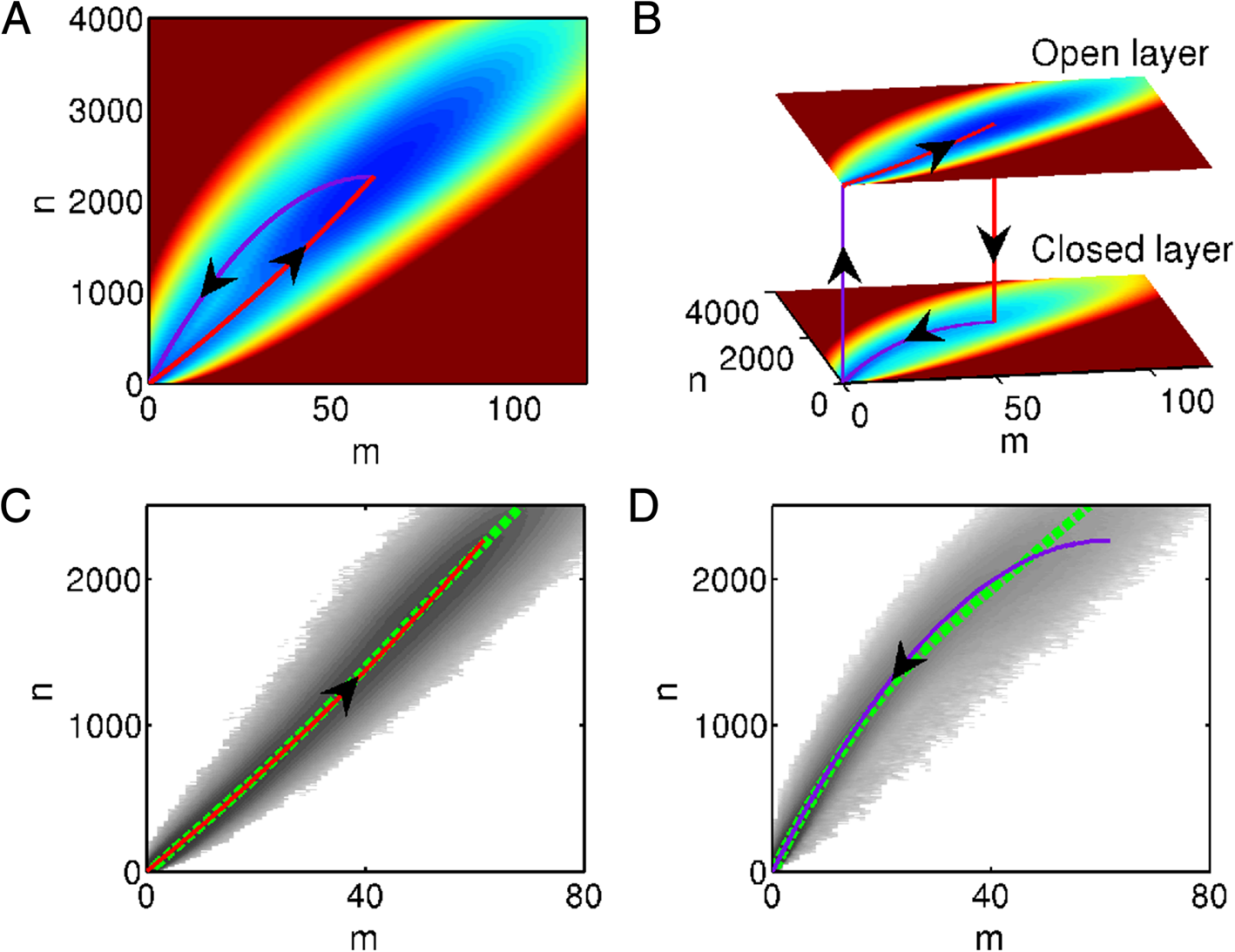
Illustrations of the energy landscapes and transition paths with slow switching rates. **a** A global view of the quasi-potential energy landscape of the genetic switching system. Switching paths from off to on state (purple solid curve) and from on to off state (red solid curve) are deterministic paths between two stable fixed points. We call the steady state with relatively large number of proteins as *on* state, whereas the steady state with relatively small number of proteins is *off* state. **b** The two-layer decomposition of subfigure A. Jumps between two layers are rare events compared with reactions on each individual landscape. **c**, **d** Averaged switching trajectories from simulations. We take the two stable fixed points in the deterministic dynamics as the starting and ending points. Darkness of the shading points represents the average duration time on reactive trajectories. We keep track of the trajectories from the time that gene states change to the time that the system reaches new stable state. We use principal curve to characterize the averaged switching trajectories (green dashed curves). The results suggest that the average switching paths are along deterministic paths. The parameters are *K*_*R*_=100, *K*_*R*0_=0.1, *k*_*P*_=51.5, *d*_*R*_=0.7, *d*_*P*_=1.4, *d*_*G*_=0.0014, *k*_*G*_=0.028, *k*_*G*0_=0.00028, and *K*=3000

We first consider the slow regime where the gene states change very slowly compared with the synthesis and degradation of proteins and mRNAs. We start from constructing the energy landscape, which is a useful way to illustrate the dynamics of the system. We simply take the definition of the potential as *U*(*m*,*n*)=− ln*P*(*m*,*n*), which is commonly used by Wang et al. [14, 28, 29]. Here *P* is the invariant distribution with the form *P*(*m*,*n*)=*P*_0_(*m*,*n*)+*P*_1_(*m*,*n*). Solving the CME of the system (1)-(2) with brutal force, we get the energy landscape as shown in Fig. 2a. It contains two attracting basins, among which the one with large number of molecules is related to the open state, whereas the other one located at the origin is related to the closed state. As the global energy landscape reflects the steady state distribution of the system, it can not tell us non-equilibrium information. Below we will address this issue by reducing the dynamics and revealing the essential nature of the system.

A careful observation shows that the gene will stay on a single state for a very long time and then make transition to another state. So we decouple the landscape into two separated layers according to the gene state (Fig. 2b). The upper layer stands for the open state, whereas the lower layer stands for the closed state. Transitions between two layers do not often occur. Each layer has only one steady state, which can be found through deterministic rate equations

(3) $$ \begin{aligned} \frac{\mathrm{d}m}{\mathrm{d}t}= &\alpha\left(k_{R0}+k_{R}\frac{n}{n+K}\right)-d_{R}m,\\ \frac{\mathrm{d}n}{\mathrm{d}t}= &k_{P}m-d_{P}n, \end{aligned}  $$

where *α* takes value 0 if the gene is closed and value 1 if the gene is open. The steady states of Eq. 3 when *α*=0 and 1 characterize the attraction points in the overall energy landscape and the relaxation from one steady state to the other corresponds to the transition paths. If the noise effect can not be neglected, we can further get the diffusion approximation beyond Eq. 3 (see Additional file 1 for details). When the system size is large, the dynamical process on each level can be approximated by diffusion process [30–32]. We consider the concentration variables *x*=*m*/*V*, *y*=*n*/*V* on the lattice $\mathbb {N}^{2}/V$, where *V* is the volume size. Define ***x***=(*x*,*y*), then the first and second moments of the process satisfy

(4) $$ \begin{aligned}  \dot{\boldsymbol{x}}=&\;\boldsymbol{c}(\alpha,\boldsymbol{x}(t)),\\ \dot{\boldsymbol{\sigma}}=&\;\boldsymbol{\sigma}(t)\boldsymbol{J}^{T}(\alpha,t)+\boldsymbol{J}(\alpha,t)\boldsymbol{\sigma}(t)+2\boldsymbol{D}(\alpha,\boldsymbol{x}(t)), \end{aligned}  $$

where ***c***(*α*,***x***) and ***D***(*α*,***x***) have the form

(5) $$ \begin{array}{l} \boldsymbol{c}=\left[\begin{array}{c} -d_{R} x+\alpha\left(k_{R0}+k_{R}\frac{y}{y+k}\right)\\ k_{P} x-d_{P}y \end{array}\right],\\ \boldsymbol{D}=\frac{1}{2}\left[ \begin{array}{cc} d_{R} x+\alpha\left(k_{R0}+k_{R}\frac{y}{y+k}\right) &0\\ 0& k_{P}x+d_{P}y \end{array}\right]. \end{array}  $$

***J***(*α*,*t*) is the Jacobian of ***c***(*α*,***x***(*t*)), and ***σ***(*t*) is the covariance matrix of the system. Thus, given *α*=0 or 1, the dynamics of the system can be well approximated by the time evolution of the moment Eqs. 4 and the transition paths mainly follow the solution of the ODEs (3) (Fig. 2a, b). This is verified by Gillespie’s stochastic simulation algorithm and the results are shown in Fig. 2c, d. The darkness of the shading points represents the number of visits for transition paths, and the fitted curve shows nice coincidence with the deterministic path, which strongly supports our methodology.

This understanding can also be utilized to get a simple approximation of the invariant distribution. When the rates of genetic switches are extremely slow, the system will mainly stay in closed-state basin or open-state basin with rare transitions. So we have *κ*≈0, and we can approximate the steady state distribution $P^{ss}_{\alpha }$ by simple Gaussian distribution $N(\boldsymbol {x}_{\alpha }^{ss},\boldsymbol {\sigma }_{\alpha }^{ss})$, where $\boldsymbol {x}_{\alpha }^{ss}$ and $\boldsymbol {\sigma }_{\alpha }^{ss}$ are the corresponding steady state solution of Eq. 4. Furthermore we can combine the two distributions to depict the invariant distribution of the whole system through a Gaussian mixture model

(6) $$ P^{ss}(\boldsymbol{x})\approx wN(\boldsymbol{x}_{0}^{ss},\boldsymbol{\sigma}_{0}^{ss})+(1-w)N(\boldsymbol{x}_{1}^{ss},\boldsymbol{\sigma}_{1}^{ss}),  $$

where the mixture weight *w* is the proportion of time that the system stays on the closed layer. Since the mean time that gene stays on the open layer is $d_{G}^{-1}$, and the mean time that gene stays on the closed layer is approximately $k_{G0}^{-1}$ (the steady state *O*_*P*_=0 on this layer), the value of *w* satisfies

(7) $$ \frac{w}{1-w}\approx \frac{k_{G0}}{d_{G}}.  $$

**Fig. 3:**
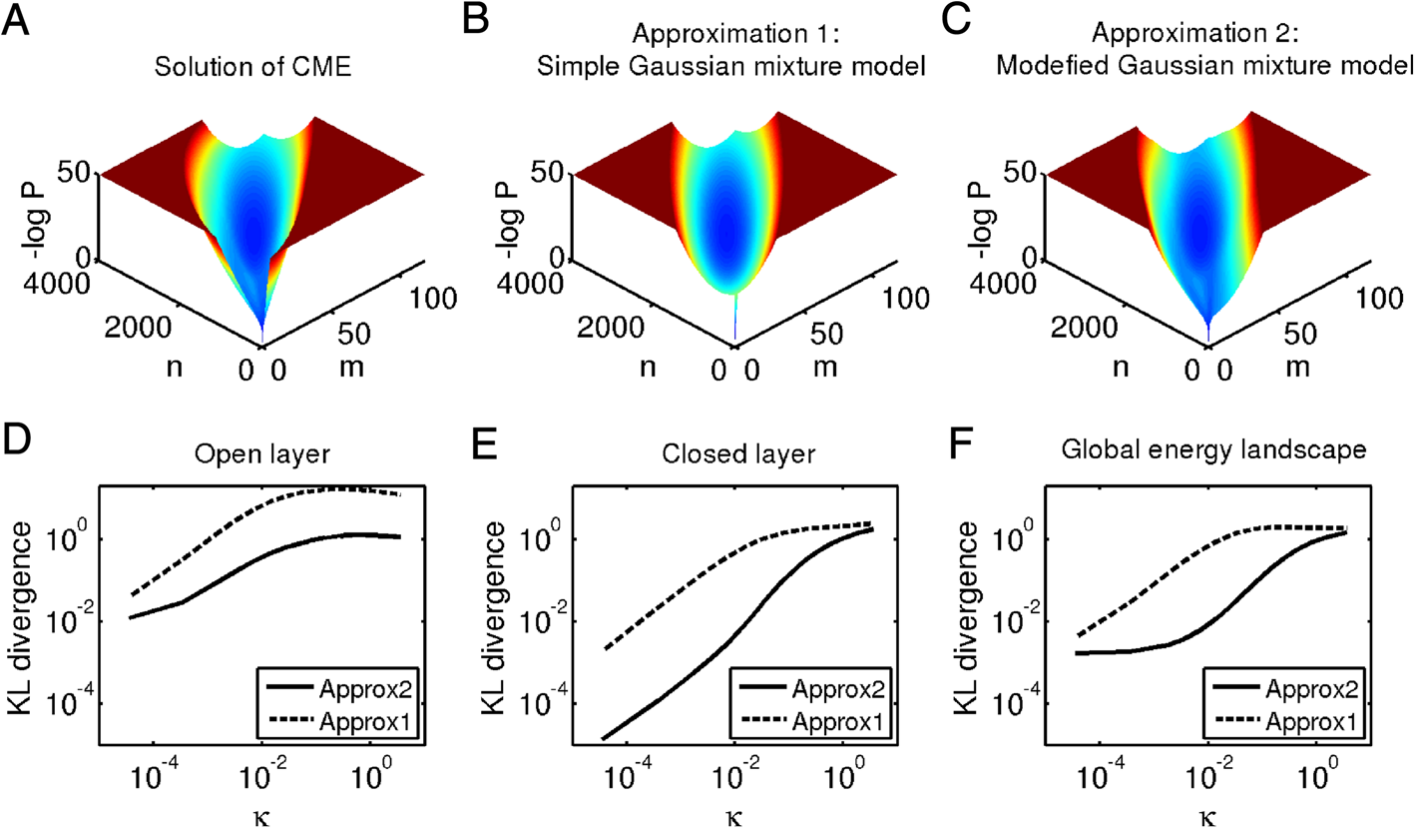
Illustrations of the simple Gaussian mixture approximation and the modified Gaussian mixture approximation to the accurate energy landscape in the regime of slow switching rates. **a** The solution of CME which we take as the accurate energy landscape. **b** Illustration of the simple Gaussian mixture approximation. **c** Illustration of the modified Gaussian mixture approximation. **d**, **e**, **f** The relative errors of two approximation methods on the open layer, closed layer and global energy landscape. Dashed lines correspond to the simple Gaussian mixture approximation and solid lines correspond to the modified approximation. We use the KL divergence (i.e. Kullback-Leibler divergence) to measure the difference between two approximations and the accurate solution of CME. To make the KL divergence well defined, we assume that the minimum value of the distributions in the truncated domain is 10^−16^ (i.e. replace the values which are less than 10^−16^ with 10^−16^). From the quantitative comparisons, we find that the modified approximation is superior than the simple approximation since more detailed information are encoded. Parameter values are the same with those in Fig. 2

Solve it and we get *w*=*k*_*G*0_/(*k*_*G*0_+*d*_*G*_). This simple Gaussian mixture approximation is shown and compared with the accurate distribution in Fig. 3a, b. The approximation seems to fit the solutions of Eqs. 1-(2) well, especially around the steady point. But some details corresponding with the switching paths are neglected, which makes some difference with the accurate solutions.

When the rates of genetic switches are relatively slow but not extremely slow, we can refine the approximation (Eq. 6) by taking into account the transition path information. On the open layer, we solve Eq. 4 with initial values $(\boldsymbol {x}_{0}^{ss},\boldsymbol {\sigma }_{0}^{ss})$ to get solutions ($\boldsymbol {x}_{1}^{t},\boldsymbol {\sigma }_{1}^{t}$). Then we use the time-average of Gaussian distributions to approximate $P_{1}^{ss}(\boldsymbol {x})$, i.e.

(8) $$ P_{1}^{ss}(\boldsymbol{x})\approx \frac{1}{T_{1}}\int_{0}^{T_{1}} p(\boldsymbol{x},t)\mathrm{d}t,  $$

where *p*(***x***,*t*) is the probability distribution density of $N(\boldsymbol {x}_{1}^{t},\boldsymbol {\sigma }_{1}^{t})$, and *T*_1_ is the average time that system keeps staying on the open layer. The same approach applies to the approximation of $P_{0}^{ss}(\boldsymbol {x})$. Then we combine $P_{0}^{ss}(\boldsymbol {x})$ and $P_{1}^{ss}(\boldsymbol {x})$ to get

(9) $$ P^{ss}(\boldsymbol{x})\approx wP_{0}^{ss}(\boldsymbol{x})+(1-w)P_{1}^{ss}(\boldsymbol{x}),  $$

where the value of *w* remains the same. We call Eq. 9 the modified Gaussian mixture approximation and compare it with the accurate distribution in Fig. 3c. Indeed, Eq. 6 is the limit of Eq. 9 when *κ*→0.

We also compute the relative errors of the two approximations and find that the modified Gaussian mixture approximation is superior than the simple Gaussian mixture approximation nearly in the whole slow regime (Fig. 3d, e, f). On both the open and the closed layers, the modified Gaussian approximation performs better than the simple Gaussian approximation. When the switching rates are extremely slow, the two approximations perform equally well. When *κ* is not very small but still belongs to the slow regime, the simple Gaussian approximation on the closed layer which is a single-point delta distribution, deviates far from the accurate solution. This is the main reason that simple Gaussian mixture approximation has relatively large errors.

### Case B: fast regime

We now consider the case that genetic switches are much faster than the other reactions in the system. In this regime, the effective synthesis rate of mRNA is (*k*_*R*_*O*_*P*_+*k*_*R*0_)*k*_*on*_/(*k*_*on*_+*k*_*off*_) according to the QSSA, where *k*_*on*_ and *k*_*off*_ stand for the gene activation and inactivation rates. The deterministic mean-field description of this model yields the ODEs:

(10) $$ \begin{aligned} \frac{\mathrm{d}m}{\mathrm{d}t}&= \frac{\left(k_{R0}+k_{R}\frac{n}{n+K}\right)\left(k_{G0}+k_{G}\frac{n}{n+K}\right)}{\left(d_{G}+k_{G0}+k_{G}\frac{n}{n+K}\right)}-d_{R}m,\\ \frac{\mathrm{d}n}{\mathrm{d}t}&= k_{P}m-d_{P}n. \end{aligned}  $$

This system has two stable fixed points and one saddle in physically reasonable parameter regime. These two stable fixed points correspond to the expressed and unexpressed states at which the copy number of proteins is at high or low state, respectively. As shown in the previous section, the global energy landscape of the slow regime also has two basins, which looks like the fast case in the first sight. But the essential nature is totally different. The bistability of the system in the slow regime originates from the simple two-layer dynamics induced by genetic switching, whereas the bistability in the fast regime is from an adiabatic reduction of the switching dynamics. The previous work of the authors has established a good framework to understand the metastability based on large deviation theory for Markov processes [15, 33]. In this framework, we assume that the steady state distribution *P*^*ss*^(***x***) satisfies *P*^*ss*^(***x***)∝ exp(*VS*(***x***)), where *S*(*x*) is the global quasi-potential function that we focus on, and the volume size *V* is inversely proportional to the noise strength. From WKB asymptotics [13] or the large deviation theory [15, 33], we know that *S*(*x*) satisfies the Hamilton-Jacobi equation *H*(***x***,∇*S*)=0 from the leading order analysis for the corresponding CME, where *H* is the Hamiltonian of the system. However, solving the Hamilton-Jacobi equation is not an easy task, and we utilize an alternative efficient optimization approach to obtain *S*(***x***). We define the local quasi-potential *S*(***x***;***x***_0_) with respect to a metastable state ***x***_0_ as

(11) $$ S(\boldsymbol{x};\boldsymbol{x}_{0})=\inf_{T>0}\inf_{\phi,\phi(0)=\boldsymbol{x}_{0},\phi(T)=\boldsymbol{x}}{\int_{0}^{T}} L(\phi,\dot{\phi})\mathrm{d}t,  $$

where *L* is the Lagrangian, which is the Legendre transform of the Hamiltonian *H*, and the path *ϕ* are all possible continuous connections from ***x***_0_ to ***x*** within time *T*. From classical mechanics, the double minimization problem (11) can be transformed to a single minimization with respect to the intrinsic representation of the curves *ϕ*(*s*) by Maupertuis’ principle [15, 34] as

(12) $$ S(\boldsymbol{x}; \boldsymbol{x}_{0}) = \inf_{\stackrel{\phi(0)=\boldsymbol{x}_{0},\phi(1)=\boldsymbol{x}}{H(\phi,\boldsymbol{p})=0, H_{\boldsymbol{p}}(\phi,\boldsymbol{p})=\lambda \phi'}}{\int_{0}^{1}} p(s) \cdot d\phi(s),  $$

where *p*(*s*) and *λ* are determined from the constraints for each *ϕ*(*s*). This minimization problem can be efficiently solved by using the geometric minimum action method (gMAM) [15, 34]. *S*(***x***) can be obtained from its local version *S*(***x***;***x***_0_) by a suitable sticking procedure [15, 33]. With this approach, we can construct the global quasi-potential energy landscape *S*(***x***) and obtain the most probable transition paths between two metastable states. More details can be referred to [15].

**Fig. 4:**
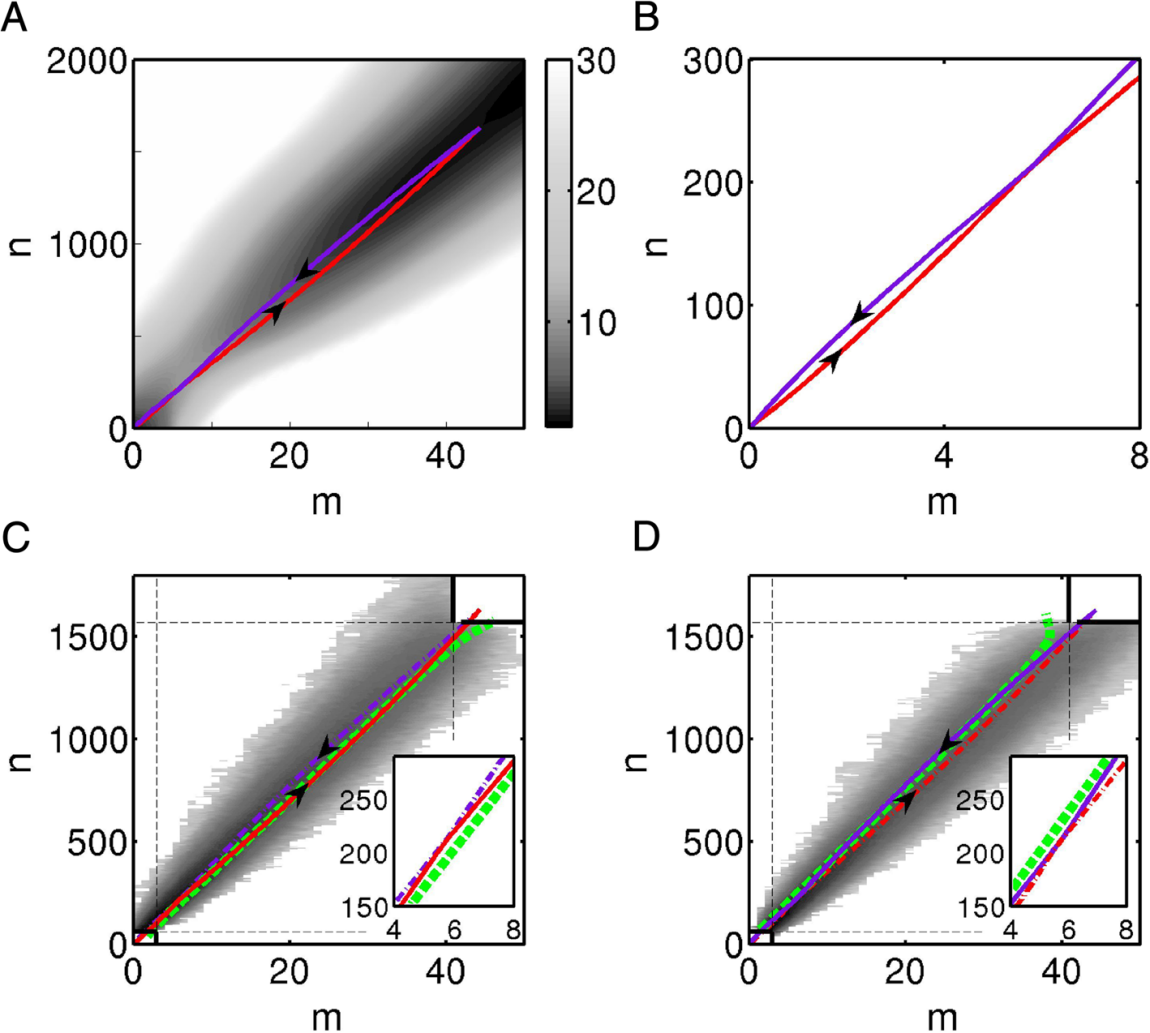
Illustrations of the energy landscapes and transition paths with fast switching rates. **a** The global quasi-potential energy landscape of the genetic switching system. The most probable switching paths from off to on state (purple solid curve) and from on to off state (red solid curve) both pass through the saddle point. **b** Details of subfigure A. **c**, **d** The principal curves (green dashed curves) fitted from shading points obtained by Gillespie’s algorithm compare with the optimal transition paths. The result shows good matching between the fitted curves and the theoretical results. The parameters *d*_*G*_=140, *k*_*G*_=2800, *k*_*G*0_=28, and the others are the same with those in Fig. 2

With proper values of parameters, the system has two metastable states and the global quasi-potential energy landscape can be constructed as shown in Fig. 4a. The landscape looks similar with Fig. 2, but transition paths between two basins show different features. In the slow regime, the transition paths are characterized by the solutions of deterministic ODEs (3) and they do not intersect at any point. In the fast regime, the most probable transition paths of on-to-off switching and off-to-on switching are not identical, either. They intersect at an unique point, i.e. the saddle point of the deterministic ODEs (10), but they do not cross over each other (Fig. 4a, b). We also present the results by Gillespie’s algorithm and compare the theoretically predicted optimal transition paths and the fitted curves in Fig. 4c, d. The comparison shows good matching between the fitted curves and the theoretical transition paths. Our results are consistent with the previous studies [15, 35].

### Case C: intermediate regime and comparisons

In both the fast and slow regimes, we have already illustrated some analytical approaches to quantitatively understand the global energy landscape and transitions between metastable states in gene expression model with positive feedback. But in the intermediate regime, where all of the reactions are on the same time scale, these analytical methods fail, since it is difficult to find the appropriate deterministic equations and hard to know whether steady states exist in the system. Thus we compute the key properties in the intermediate regime through Gillespie’s algorithm and compare the results with the slow and fast regimes.

**Fig. 5:**
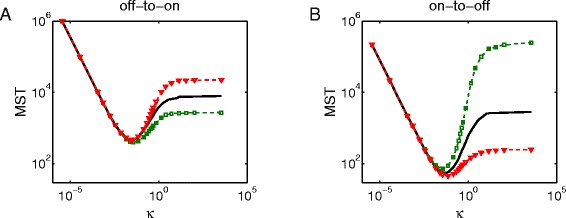
The mean switching time (MST) as a function of *κ* with different positive feedback strength. **a** MST curve of off-to-on switch. **b** MST curve of on-to-off switch. Parameters are the same with with Fig. 2 except *d*_*G*_, *k*_*G*_, *k*_*G*0_, and *K*. When *κ* changes, *d*_*G*_ should change simultaneously to keep *d*_*G*_/*k*_*G*_, *d*_*G*_/*k*_*G*0_ locked (these ratios are the same with Fig. 2). We use different strength of positive feedback for simulations, which is *K*=2754 (green dashed lines with square markers), 3000 (black solid lines), 3211 (red dashed lines with triangular markers). All of the results are obtained from Gillespie’s algorithm

We simulate the mean switching time (MST) with different parameters. The MST is the average transition time between two basins on the global energy landscape, which is often used to characterize the stability of the attractors. Figure 5 shows the MST with respect to different *κ* and positive feedback strength *K*. When *κ* increases from 10^−5^ to 10^5^, the gene activation rate changes from slow to fast regimes. Since we focus on switching times between metastable states, the value of *K* should be chosen to guarantee that bistability always exists in the system with respect to all the possible values of *κ*. We define the existence of bistability as that the system stay at each metastable state with probability more than 0.01. In the slow regime, bistability always exists for any positive *K*. In the intermediate and fast regimes, we find two boundary values of *K*, which are *K*=2754 and *K*=3211. When *K*<2754, the system will stay at the off state with probability less than 0.01, whereas when *K*>3211, the system will stay at the on state with probability less than 0.01. The smaller *K* is, the larger positive feedback strength is.

In the slow regime, the MST can be characterized by the waiting time for transitions to occur, so the MST decreases as *κ* increases. In this regime, *K* has little effect on MST, which is consistent with our analysis in Case A that the MST of on-to-off and off-to-on transitions is approximately $d_{G}^{-1}$ and $k_{G0}^{-1}$, respectively. When *κ* increases to a value between 10^−2^ and 10^−1^, the MST decreases to its global minimum point, and a neighborhood of this point is corresponding to the intermediate regime. Then the MST goes up with *κ* increasing, and finally reaches a stable value in the fast regime. In the fast regime, when *K* changes from 2754 to 3211 (i.e. the strength of positive feedback decreases), the MST of off-to-on switch increases by a factor of about 10, whereas the MST of on-to-off switch decreases by a factor of about 10^3^. Thus, if the positive feedback becomes stronger, the system will be more probable to stay at the on state, with shorter off-to-on switching time and longer on-to-off switching time.

Longer MST indicates that metastable states are more stable. Thus, our results in Fig. 5 show that the slow and fast regimes perform better in stability, whereas in the intermediate regime, genetic switches, transcription and translation are on the same time scale, which contributes to large fluctuations of molecule numbers and relatively short MST. These properties remain the same with different positive feedback strength. Furthermore, we also did some interesting simulations to study the change of transition paths as a function of *κ* (see Additional file 1 for details).

We should mention that the above points are direct observations from numerical results, which are hard to be proven analytically. However, we can confirm from the derivations in Case A that when *κ* is extremely small, the MST of on-to-off and off-to-on transitions is approximately $d_{G}^{-1}$ and $k_{G0}^{-1}$. That is to say, the downward-sloping shapes of the left parts of the MST curves are qualitatively robust. Besides, when *κ* goes to infinity, the MST will converge to some constant values [15]. So the flat parts on the right of the MST curves are also theoretically supported. The existence of “valleys” in the MST curves is in accordance with the intuition, but it is difficult to give a rigorous proof. To investigate the robustness of the whole qualitative shape of the MST curve with respect to different feedback actions, we simulated all the results with *O*_*P*_=*n*^2^/(*n*^2^+*K*^2^), and found that the shapes of the MST curves are almost the same as in Fig. 5. We may reasonably infer that all results still hold assuming that *O*_*P*_ is a Hill function and the bistability exists in both slow and fast regimes (see Additional file 1 for more details). These results suggest a natural methodology to distinguish the different genetic activation/inactivation rates in the cellular phenotype observations, which is described in detail in the continued text.

### Distinguishing the three regimes

In molecular experiments, it is hard to directly measure the switching rates of the active or inactive gene state. Based on the obtained results, we propose a method to distinguish which regime the considered system falls in. Consider a gene expression circuits with the positive feedback in a eukaryotic cellular system (Fig. 1), such as the yeast or the immune cell etc, we can observe the level of proteins through fluorescence or other markers by flow cytometry. Below we will show how to take advantage of these perceivable data and related analysis to make corresponding distinction.

First let us introduce how to identify the intermediate regime. We utilize the strength of cellular memory as the index, and more concretely, we sort the cells by the protein level and observe how long the sorted cells will take to reach the original invariant distribution of proteins. The detailed flow cytometry procedure of our experiments *in silico* is stated in the following two steps.

**Fig. 6:**
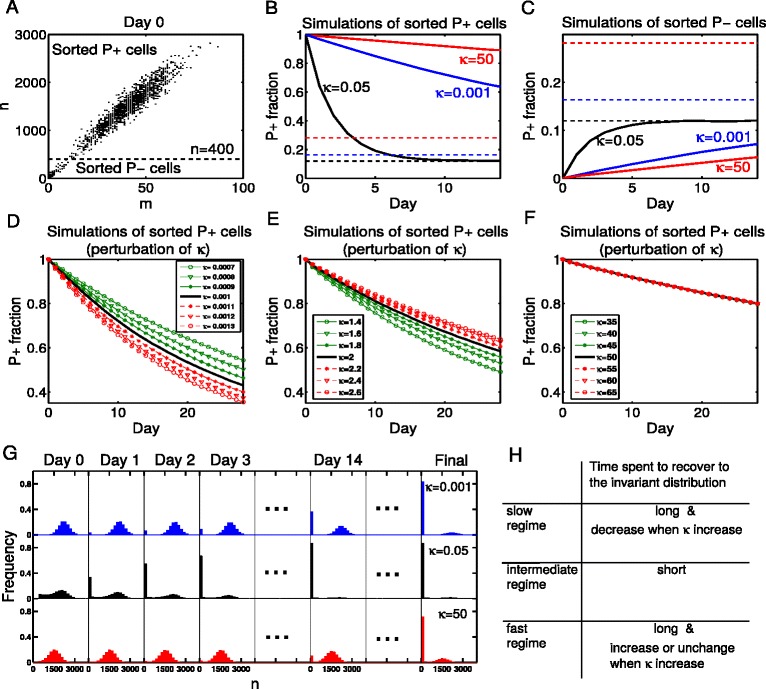
A method we propose to distinguish the slow, intermediate and fast regimes. (**a**) 50,000 sample points are selected randomly according to the invariant distribution solved from the CME, i.e. 50,000 cells are selected randomly from a cell population. Then the cells are classified by the number of proteins. We call the cells with large number of proteins (*n*>400) as *P+ cells*, and the cells with small number of proteins (*n*≤400) as *P- cells*. We define the fraction of P+ cells in a cell group as P+ fraction. **b**, **c** We simulate the evolution of the two groups for a long time with Gillespie’s algorithm. The change of P+ fractions are recorded in solid lines and the final values of P+ fractions are drawn in dashed lines. It is obvious that cells of the intermediate regime have shorter memory, for they rapidly recover to the origin unsorted state (invariant distribution). **d**, **e**, **f** If we perturb *κ* (through changing *d*_*G*_ and keeping the ratios *d*_*G*_/*k*_*G*_, *d*_*G*_/*k*_*G*0_ and other parameters unchanged), the lines of P+ fractions are shifted in different ways with respect to different regimes. In the slow regime (*κ*=0.001), P+ fraction changes more quickly when *κ* increases, whereas in the relative fast regime (*κ*=2, 50), the time spent for cells to recover to the invariant distribution is longer or unchanged when *κ* increases. **g** The evolution of distributions in the view of protein molecules. These are detailed data of subfigure B which illustrate our thought clearly. **h** Comparison of the slow, intermediate and fast regimes. Using this method, we successfully distinguish the three regimes. The parameters are the same with Fig. 5 in all the figures above
- Step 2: Culturing the sorted *P+* and *P −* groups of cells separately, and recoding the changes of *P+* fractions in each group. Figure 6b, c show the simulation results of the *P+* and *P −* groups respectively. The evolution of the protein distributions of the sorted *P+* group is simulated and illustrated in Fig. 6g, including results of the slow, intermediate and fast regimes. The sorted *P+* group of cells in the intermediate regime can be observed to evolve quickly to its initial equilibrium state. In the third day, a large percent of *P+* cells in the intermediate regime have evolved to the *P −* group, whereas the *P+* cells in the slow and fast regimes have little change. This suggests that cellular memory is much stronger in the slow and fast regimes compared with the intermediate regime.

- Step 1: Sorting the cells by the protein levels. For example, we choose *n*=400 as the threshold of classification in our model setup, where *n* is the number of protein molecules in a cell. We call the cells with large number of proteins (*n*>400) as *P+ cells*, and the cells with small number of proteins (*n*≤400) as *P − cells*. We define the fraction of *P+* cells in a cell group as the *P+ fraction*. Figure 6a illustrates the numerically sorted results of the system, including 50,000 sample points which are randomly selected according to the invariant distribution, and *P+/P −* cells are classified by the protein level in each cell. To apply this approach to the gene regulatory network with different parameters, one need to choose an appropriate threshold according to the real situation, which needs to lie between two metastable states.

In the real experiments, we do not know whether the time for an appreciable change of protein levels is long or short without reliable reference. But the MST in the intermediate regime is approximately the time for cells to reach a metastable state after leaving another metastable state, thus we can use it as a reference time. We define *T* as the on-to-off (or off-to-on) transition time, which does not contain the dwell time at the on (or off) state. Since the synthesis and degradation rates of mRNAs and proteins can be estimated through experimental approaches, we can theoretically figure out the time that the system will spend to reach the off state after leaving the on state. That is to say, *T* can be roughly estimated a priorily. So we make experiments like Fig. 6g for a period of time which is close to *T*. If the value of *P+* fraction changes evidently towards the equilibrium level, we will know that the system belongs to the intermediate regime.

Second we try to identify the fast and slow regimes through the perturbation-response behavior of changing *κ*. As shown in Fig. 5a, the MST becomes larger and larger when *κ* approaches to zero, but it increases and gradually stays close to a fixed value when *κ* is large. Thus, if we perturb *κ* through injection of particular chemicals, cells in the two regimes will perform differently in the experiments we designed above. Figure 6d, e, f illustrate the results of corresponding numerical simulations. In the slow regime (*κ*=0.001), *P+* fraction changes more quickly when *κ* increases, whereas in the relatively fast regime (*κ*=2, 50), the time for cells to recover to the invariant distribution is longer or unchanged when *κ* increases. With this perturbation-response approach, the slow and fast regimes can also be distinguished from each other (Fig. 6h). When the positive feedback strength *K* takes the boundary values mentioned in case C, the simulation experiments above still work well (see Additional file 1 for details). This proposal, based on our theoretical understanding of the feature of the three regimes, is yet to be justified by experimentalists.

## Discussion

Understanding the dynamical features and the energy landscapes of the gene regulatory network is an interesting and challenging topic in quantitative biology. Some approaches focused on the regime of fast switching rates have been developed in recent years, whereas the slow regime which many eukaryotic cells correspond to attracts less attention. In this paper, besides the theoretical analysis of the essential dynamics in the slow, intermediate and fast regimes, we provide a method to distinguish these three regimes. The result is meaningful and enlightening for the design of corresponding biological experiments.

With regard to a previous work about stochastic dynamics of single regulating genes [19], we provide a systematic study to understand the genetic switching dynamics when the switching rates are in different regimes. Although the global quasi-potential energy landscape and other properties of the system can be obtained from brutal force through numerical simulations, the theoretical reductions can reveal the intrinsic mechanisms. We analyze the model theoretically in the slow and fast regimes and successfully reconstruct the global energy landscape which reflects the inherent pattern of system behavior. But we obtain little theoretical results in the intermediate regime, since the nonexistence of time-scale separation makes this problem more challenging. Recent new ideas proposed in this field may be helpful in the study of this regime [36, 37].

A related but different concept with multi-layer landscapes for the DNA fast switching case was mentioned in a recent literature [36]. However, we are mainly concerned with the two-layer case when the system is in slow regime. Besides understanding the system as decoupled layers, we further study the properties about transition paths and average switching time, and we construct the global potential energy landscape through simple Gaussian mixture and modified Gaussian mixture approximations. These pictures are quite different from the case of fast regime.

The MST is a key characterization of gene regulatory networks and has been studied analytically with respect to different genetic switching rates in [38]. Compared with the detailed theoretical analysis for a typical one-dimensional self-activating switch model, we can not give fully theoretical results in the fast regime, i.e. the strictly adiabatic regimes considered in [38], since our model involves both mRNA and protein explicitly. However, we present efficient numerical methods to make the quantitative estimates. The results for the extremely slow regime are also consistent with those obtained for non-adiabatic regime (i.e. Eq. 7). We also suggested the landscape as an intuitive tool to understand the essential dynamics of our model. Overall, the shape of the MST curve we obtained as a function of *κ* is qualitatively similar as in [38] as well.

The proposed quasi-potential energy function *S*(***x***) in the fast regime has close connection with the potential function considered in previous studies. In Wang et al’s approach [14, 39], they proposed to define the energy landscape as *U*(***x***)=− ln*P*^*ss*^(***x***), where *P*^*ss*^(***x***) is the steady state distribution. We have the connection that $S(\boldsymbol {x})={\lim }_{\textit {V}\rightarrow \infty } V^{-1}U(\boldsymbol {x})$ [15, 40] since *U*(***x***) itself depends on the system size *V*. We also note that the proposed quasi-potential *S*(***x***) satisfies the same Hamilton-Jacobi equation as the potential defined in [41, 42] in the diffusive approximations. But in addition, we provided a variational minimization approach to explicitly construct the *S*(***x***). Nonetheless, this construction is only valid for the fast regime and it is quite different from the picture we showed in the slow regime.

Although our four-state genetic switching model is based on chromatin remodeling, this is no longer expressed in the final simplified two-state model. In fact, some prokaryotic cells also have similar gene regulatory networks like Fig. 1b [17]. Our study mainly focuses on the two-state model, which can be suitable for a wide variety of cellular systems rather than the motivating chromatin remodeling process. Our simulation experiment to distinguish the slow, intermediate and fast regimes is an important point of the paper. So far, we are not able to perform real experiments to make the verification. The real experimental systems may be also more complicated than the model considered here. But our simulation experiment is logically true and self-consistent, and there have been related experiments performing well in Th2 cells [16]. Finding suitable system and validating our proposal will be an interesting task in the future.

Our study has potential application in single-cell experiments [43–45]. In the single-cell gene expression analysis, the protein level of each cell at different time points can be obtained through tracking and recording the behavior of each cell by fluorescence microscopy. With these data, one can obtain the switching time as the time difference between two consecutive states of a cell. Thus the MST can be roughly estimated. Similar as the procedure in flow cytometry experiments, the information about MST can be utilized to identify the intermediate regime, and the fast and slow regimes can be distinguished by perturbing *κ*. However, these points remain to be investigated in detail in the follow-up researches.

## Conclusions

In this paper, we proposed a methodology to distinguish the regime of genetic switching rates from phenotypic variations in a simulation experiment. To accomplish this task, we put forward different reduction and investigation methods to reveal the essential dynamics of the genetic switching system when the switching rates are relatively slow, fast, or medium compared with the degradation rates of proteins. Although in the fast and slow regimes, the observed steady state distributions of the mRNAs and proteins are similar, we showed that the underlying mechanisms are qualitatively different. Based on our results, we provided a simulation experiment to distinguish these three regimes. The intermediate regime can be identified from the fact that the strength of cellular memory is lower than the other two cases, and the fast and slow regimes can be distinguished by their different perturbation-response behavior with respect to the switching rates perturbations. We also discussed the robustness of our results with respect to different choices of parameters and feedback function forms. This study provides insights to the experimental analysis of the gene expression system. It will be interesting to investigate similar problems in different experimental setups in the future.

## Additional file

Additional file 1
**Supporting information.** Simplification of the four-state genetic switching model; Gaussian approximation in the regime of slow switching rates; An extension of the results: $O_{P}=n^{k}/(n^{k}+K^{k}),\; k\in \mathbb {N}$; Distinguishing the three regimes when the positive feedback strength *K* takes the boundary values; The change of switching paths as a function of *κ*; and biological relevance of the parameter values.

## Abbreviations

MST: Mean switching time
QSSA: Quasi-steady-state approximation
CME: Chemical master equation
gMAM: Geometric minimum action method
