# Supplementary material for: Distinguishing the rates of gene activation from phenotypic variations
Source: BMC Syst Biol. 2015 Jun 18;9:29. doi: 10.1186/s12918-015-0172-0 (PMC4479085; doi:10.1186/s12918-015-0172-0)
Supplement: Additional file 1 — Supporting information. Simplification of the four-state genetic switching model; Gaussian approximation in the regime of slow switching rates; An extension of the results: $O_{P}=n^{k}/(n^{k}+K^{k}),\; k\in \mathbb {N}$OP=nk/(nk+Kk),k∈ℕ; Distinguishing the three regimes when the positive feedback strength K takes the boundary values; The change of switching paths as a function of κ; and biological relevance of the parameter values. [file 12918_2015_172_MOESM1_ESM.pdf]

Distinguishing the Rates of Gene Activation from Phenotypic Variations: Supporting Information

Ye Chen, Cheng Lv, Fangting Li and Tiejun Li

Content

I. Simplification of the four-state genetic switching model

II. Gaussian approximation in the regime of slow switching rates

III. An extension of the results: $O_P = n^k/(n^k + K^k),\ k \in \mathbb{N}$

IV. Distinguishing the three regimes when the positive feedback strength K takes the boundary values

V. The change of switching paths as a function of κ

VI. Biological relevance of the parameter values

Simplification of the four-state genetic switching model

For the four-state genetic switching model, the chemical master equation has the form

$$
\begin{aligned}
\frac{d}{dt}P(1,m,n;t) =& -k_F nP(1,m,n;t) + d_F P(2,m,n;t) - k_{G0}P(1,m,n;t) + d_G P(3,m,n;t) + \mathbf{A}P(1,m,n;t),\\
\frac{d}{dt}P(3,m,n;t) =& -k_F nP(3,m,n;t) + d_F P(4,m,n;t) - d_G P(3,m,n;t) + k_{G0}P(1,m,n;t)\\
&+ k_{R0}(\mathbf{E}_m^{-1}-1)p(2,m,n;t) + \mathbf{A}P(3,m,n;t),\\
\frac{d}{dt}P(4,m,n;t) =& k_F nP(3,m,n;t) - d_F P(4,m,n;t) - d_G P(4,m,n;t) + k_{G1}P(2,m,n;t)\\
&+ k_{R1}(\mathbf{E}_m^{-1}-1)p(3,m,n;t) + \mathbf{A}P(4,m,n;t),\\
\frac{d}{dt}P(2,m,n;t) =& k_F nP(1,m,n;t) - d_F P(2,m,n;t) - k_{G1}P(2,m,n;t) + d_G P(4,m,n;t) + \mathbf{A}P(2,m,n;t),
\end{aligned}
\tag{S1}
$$

where $\mathbf{A}$ is the associated translation and degradation operator defined as

$$
\mathbf{A}P(\alpha,m,n;t) = d_P(\mathbf{E}_n^1-1)[nP(\alpha,m,n;t)] + d_R(\mathbf{E}_m^1-1)[mP(\alpha,m,n;t)] + k_P(\mathbf{E}_n^{-1}-1)P(\alpha,m,n;t), \tag{S2}
$$

and $P(\alpha,m,n;t)$ stands for the probability that there are m mRNA molecules and n protein molecules in the system at time t when gene is closed and unbounded ($\alpha = 1$), closed and bounded ($\alpha = 2$), open and unbounded ($\alpha = 3$), or open and bounded ($\alpha = 4$). Here we assume that the number of protein molecules is large in the system, and binding/release of regulatory proteins is fast compared with all the other reactions. So we have $n \gg 1$ and k_F, d_F is much bigger than the other parameters. Thus, binding and release of regulatory proteins can be approximately written as

$$
\mathrm{DNA}_{\text{unbounded}} + n \underset{d_F}{\overset{k_F}{\rightleftharpoons}} \mathrm{DNA}_{\text{bounded}} + n.
$$

According to the quasi-steady-state approximation (QSSA) and Equation (S1), we obtain

$$
\begin{aligned}
k_F nP(1,m,n;t) &\approx d_F P(2,m,n;t),\\
k_F nP(3,m,n;t) &\approx d_F P(4,m,n;t).
\end{aligned}
\tag{S3}
$$

Then we define

$$
\begin{aligned}
Q_0(m,n;t) &= P(1,m,n;t) + P(2,m,n;t),\\
Q_1(m,n;t) &= P(3,m,n;t) + P(4,m,n;t),
\end{aligned} \tag{S4}
$$

where $Q_\alpha(m,n;t)$ is the probability that there are m mRNA molecules and n protein molecules in the system at time t when gene is closed ($\alpha = 0$) or open ($\alpha = 1$). From Eqs. S3 and S4, we know

$$
\begin{aligned}
P(1,m,n;t) =& \frac{d_F}{k_F n + d_F} Q_0(m,n;t) \approx \frac{K}{n+K} Q_0(m,n;t),\\
P(3,m,n;t) =& \frac{d_F}{k_F n + d_F} Q_1(m,n;t) \approx \frac{K}{n+K} Q_1(m,n;t),\\
P(4,m,n;t) =& \frac{k_F n}{k_F n + d_F} Q_1(m,n;t) \approx \frac{n}{n+K} Q_1(m,n;t),\\
P(2,m,n;t) =& \frac{k_F n}{k_F n + d_F} Q_0(m,n;t) \approx \frac{n}{n+K} Q_0(m,n;t),
\end{aligned} \tag{S5}
$$

where $K = nd_F/k_F$. Thus, Equation (S1) can be simplified as

$$
\begin{aligned}
\frac{d}{dt} Q_0(m,n;t) =& -(k_{G0} + k_G \frac{n}{n+K}) Q_0(m,n;t) + d_G Q_1(m,n;t) + \mathbf{A} Q_0(m,n;t),\\
\frac{d}{dt} Q_1(m,n;t) =& (k_{G0} + k_G \frac{n}{n+K}) Q_0(m,n;t) + (k_{R0} + k_R \frac{n}{n+K})(\mathbf{E}_m^{-1} - 1) Q_1(m,n;t)\\
& - d_G Q_1(m,n;t) + \mathbf{A} Q_1(m,n;t).
\end{aligned} \tag{S6}
$$

We define $O_P = n/(n+K)$, and O_P is actually the relative occupancy of protein at DNA binding site. In this simplified two-state genetic switching model, $Q_0(m,n;t)$ corresponds to the closed gene state, which is the combination of state (i) and (ii) in the original four-state model, and $Q_1(m,n;t)$ has similar meaning. The jump rate into the open state and the transcription rate are controlled by O_P, whereas the jump rate into the closed state is not affected by the number of proteins. The essential properties and dynamics of the system do not change after simplification.

Gaussian approximation in the regime of slow switching rates

To analyze the behavior of the system on different layers, we start from studying the backward operator $\mathcal{L}$ associated with the transcription and translation process

$$
\begin{aligned}
\mathcal{L}h(0,m,n) &= \mathbf{A}^\dagger h(0,m,n),\\
\mathcal{L}h(1,m,n) &= \mathbf{A}^\dagger h(1,m,n) + (k_{R0} + k_R \frac{n}{n+K})(\mathbf{E}_m^1 - 1) h(1,m,n),
\end{aligned} \tag{S7}
$$

where h is any function of 3-tuple $(\alpha, m, n) \in \{0,1\} \times \mathbb{N}^2$, m, n represent the number of mRNAs and proteins, and $\alpha = 0, 1$ represent the inactive and active state of gene, respectively. $\mathbf{E}_n^j$ is the raising operator acting on $f(n)$ as $\mathbf{E}_n^j f(n) = f(n+j)$. Genetic switches are ignored in this regime (d_G, k_G, $k_{G0} \ll 1$). The operator $\mathbf{A}^\dagger$ is the adjoint of $\mathbf{A}$ defined as

$$
\mathbf{A}^\dagger h(\alpha,m,n) = d_P n(\mathbf{E}_n^{-1} - 1) h(\alpha,m,n) + d_R m(\mathbf{E}_m^{-1} - 1) h(\alpha,m,n) + k_P m(\mathbf{E}_n^1 - 1) h(\alpha,m,n). \tag{S8}
$$

To investigate the evolution of the concentration variables, we consider the variables $x = m/V$, $y = n/V$ on the rescaled lattice $\mathbb{N}^2/V$, where V is the system volume. Then the infinitesimal generator $\tilde{\mathcal{L}}$ on the rescaled lattice has the form

$$
\begin{aligned}
\tilde{\mathcal{L}}h(0,x,y) &= V\tilde{\mathbf{A}}^\dagger h(0,m,n),\\
\tilde{\mathcal{L}}h(1,x,y) &= V\tilde{\mathbf{A}}^\dagger h(1,m,n) + V(\tilde{k}_{R0} + \tilde{k}_R \frac{y}{y+\tilde{K}})(\mathbf{E}_m^1 - 1) h(1,x,y),
\end{aligned} \tag{S9}
$$

where the rescaled operator

$$
\tilde{\mathbf{A}}^\dagger h(\alpha,x,y) = d_P y(\mathbf{E}_n^{-1} - 1) h(\alpha,x,y) + d_R x(\mathbf{E}_m^{-1} - 1) h(\alpha,x,y) + k_P x(\mathbf{E}_n^1 - 1) h(\alpha,x,y), \tag{S10}
$$

and $\tilde{k}_R = k_R/V$, $\tilde{k}_{R0} = k_{R0}/V$, and $\tilde{K} = K/V$. Define $u(x,y) = h(0,x,y)$, $v(x,y) = h(1,x,y)$, $\epsilon = 1/V$, then we have the backward equations for the rescaled dynamics through Taylor expansion

$$
\begin{aligned}
\frac{\mathrm{d}u}{\mathrm{d}t} &= \mathcal{A}^\dagger u + O(\epsilon),\\
\frac{\mathrm{d}v}{\mathrm{d}t} &= \mathcal{A}^\dagger v + (\tilde{k}_{R0} + \tilde{k}_R \frac{y}{y+\tilde{K}})[\partial_x v + \frac{1}{2}\epsilon \partial_{xx} v] + O(\epsilon),
\end{aligned} \tag{S11}
$$

where $\mathcal{A}^\dagger$ is the continunized differentiation form of $\tilde{\mathbf{A}}^\dagger$ as

$$\begin{aligned}\mathcal{A}^\dagger v(x,y) =& d_P[-y\partial_y v(x,y) + \frac{1}{2}\epsilon y\partial_{yy}v(x,y)] + d_R[-x\partial_x v(x,y) + \frac{1}{2}\epsilon x\partial_{xx}v(x,y)] \\ &+ k_P[x\partial_y v(x,y) + \frac{1}{2}\epsilon x\partial_{yy}v(x,y)].\end{aligned} \tag{S12}$$

Define $\boldsymbol{x} = (x,y)$, and let $\mathcal{A}_0,\ \mathcal{A}_1$ be the adjoint operator of the right-hand side of Equation (S11). Ignoring the terms of $O(\epsilon)$, the equations for the pdf become

$$\begin{aligned}\frac{\partial P_\alpha(\boldsymbol{x};t)}{\partial t} =& \mathcal{A}_\alpha^* P_\alpha(\boldsymbol{x};t) \\ =& -\frac{\partial}{\partial x}[C_1(\alpha,\boldsymbol{x})P_\alpha(\boldsymbol{x};t)] - \frac{\partial}{\partial y}[C_2(\alpha,\boldsymbol{x})P_\alpha(\boldsymbol{x};t)] \\ &+ \frac{\partial^2}{\partial x^2}[\epsilon D_{11}(\alpha,\boldsymbol{x})P_\alpha(\boldsymbol{x};t)] + \frac{\partial^2}{\partial y^2}[\epsilon D_{22}(\alpha,\boldsymbol{x})P_\alpha(\boldsymbol{x};t)],\end{aligned} \tag{S13}$$

where

$$\begin{aligned}\boldsymbol{C}(\alpha,\boldsymbol{x}) &= \begin{bmatrix} C_1(\alpha,\boldsymbol{x}) \\ C_2(\alpha,\boldsymbol{x}) \end{bmatrix} = \begin{bmatrix} -d_R x + \alpha(\tilde{k}_{R0} + \tilde{k}_R \frac{y}{y+\tilde{K}}) \\ k_P x - d_P y \end{bmatrix}, \\ \boldsymbol{D}(\alpha,\boldsymbol{x}) &= \begin{bmatrix} D_{11}(\alpha,\boldsymbol{x}) & D_{12}(\alpha,\boldsymbol{x}) \\ D_{21}(\alpha,\boldsymbol{x}) & D_{22}(\alpha,\boldsymbol{x}) \end{bmatrix} = \frac{1}{2}\begin{bmatrix} d_R x + \alpha(\tilde{k}_{R0} + \tilde{k}_R \frac{y}{y+\tilde{K}}) & 0 \\ 0 & k_P x + d_P y \end{bmatrix}.\end{aligned} \tag{S14}$$

When $\epsilon \ll 1$, the probability distributions can be approximated by Gaussian distributions along the deterministic trajectory. The first and second order moment equations are

$$\begin{aligned}\dot{\boldsymbol{x}}(t) =& \boldsymbol{C}[\alpha,\boldsymbol{x}(t)], \\ \dot{\boldsymbol{\sigma}}(t) =& \boldsymbol{\sigma}(t)\boldsymbol{J}^T(\alpha,t) + \boldsymbol{J}(\alpha,t)\boldsymbol{\sigma}(t) + 2\boldsymbol{D}[\alpha,\boldsymbol{x}(t)],\end{aligned} \tag{S15}$$

where $\boldsymbol{J}(\alpha,t)$ is the Jacobian matrix of $\boldsymbol{C}[\alpha,\boldsymbol{x}(t)]$, and $\boldsymbol{\sigma}(t)$ is the covariance matrix of the system. Equation (S15) are on the lattice $\mathbb{N}^2/V$, and we can transfer them back to the lattice $\mathbb{N}^2$. We only need to replace $\tilde{k}_R,\ \tilde{k}_{R0},\ \tilde{K}$ in $\boldsymbol{C}(\alpha,\boldsymbol{x})$ by $k_R,\ k_{R0},\ K$, with the form of Equation (S15) unchanged.

An extension of the results: $O_P = n^k/(n^k + K^k),\ k \in \mathbb{N}$

In the regime of slow switching rates, the effective dynamics can be reduced to independent evolutions on two separate layers corresponding to gene activation and inactivation states. As discussed in the paper, the existence of this phenomenon is independent of the expression of O_P. Thus, in the case of $O_P = n^k/(n^k + K^k)$ where $k > 1$, the results in the slow regime will not change.

In the fast regime, the genetic switching system has two stable fixed points and one saddle with reasonable parameters when $O_P = n/(n+K)$. Here, we put forward an argument that *the system still has two stable fixed points and one saddle with suitable parameters when* $O_P = n^k/(n^k + K^k),\ k > 1$. At the end of this material we will show the proof of the argument. Thus, the numerical approaches in the fast regime still work.

In the intermediate regime, since it is hard to analyze the dynamics theoretically, we simulate the system numerically and find some interesting results based on intuitive observations and reasonable inferences. The downward-sloping shapes of the left parts and the flat shapes of the right parts of the MST curves have theoretical supports when $O_P = n/(n+K)$. The derivations we got show that the argument is still correct in the case that $O_P = n^k/(n^k + K^k)$. However, the U-shaped parts of the MST curves are difficult to be demonstrated analytically, despite that it is in accordance with intuition. We made a detailed discussion on this point at the end of the *Case C* in the paper. To further investigate this issue, we simulated the whole results with $O_P = n^2/(n^2 + K^2)$, and found that the shape of the MST curves is the same as in Figure 5. Hence, it is reasonable to state that with suitable parameters the shape of the MST curve is qualitatively robust when $O_P = n^k/(n^k + K^k),\ k \in \mathbb{N}$.

In the intermediate regime, since it is hard to analyze the dynamics theoretically, we simulate the system numerically and find some interesting results based on intuitive observations and reasonable inference as discussed in the paper. The downward-sloping shapes of the left parts and the flat shapes of the right parts of the MST curves have theoretical supports when $O_P = n/(n+K)$. The derivations above show that it is still correct in the case that $O_P = n^k/(n^k + K^k)$. Moreover, the U-shaped parts of the MST curves are difficult to be demonstrated analytically, despite that it is in accordance with intuition. Here we simulate the MST curves with $O_P = n^2/(n^2 + K^2)$ (Figure S1), and find that the shape of the MST curves is the same as in Figure 5 in the paper. Hence, it is reasonable to state that with suitable parameters the shape of the MST curves is qualitatively robust when $O_P = n^k/(n^k + K^k),\ k \in \mathbb{N}$.

Distinguishing the three regimes when the positive feedback strength K takes the boundary values

In the paper, we perform the simulation experiment to distinguish the slow, intermediate and fast regimes with a particular group of parameters. Since the positive feedback strength K is a key parameter in our model, it is necessary to study that whether the simulation experiment still works well when the value of K locates on the boundary. As mentioned in the paper, with other parameters unchanged, the lower bound of K is 2754, whereas the upper bound of K is 3211. The results of the case that $K = 2754$ are shown in Figure S2. We find that the value of P+ fraction/$P-$ fraction changes rapidly towards the equilibrium level in the intermediate regime, which is sufficient to help us identify the intermediate regime. In fact, in the case that $K = 3000$, either Figure 6B or 6C alone can be basis for judgment. But in this case, we have to take both Figure S2A and S2B into account, since the behavior of the P+ group when $\kappa = 50$ is indistinct, and small final values of P+ fraction may lead to meaningless results because of observation errors. The perturbation-response approach which aims at distinguishing the slow and fast regimes, is also very effective in this case. In the slow regime ($\kappa = 0.001$), P+ fraction changes more quickly when κ increases, whereas in the relatively fast regime ($\kappa = 2,\ 50$), the lines of P+ fractions are shifted down or unchanged when κ increases. The results of the case that $K = 3211$ are shown in Figure S3. The discussions above are also applicable to this case, and we do not repeat the details here.

The change of switching paths as a function of κ

In the slow regime, the switching paths between two metastable states are ODE paths, whereas in the fast regime, the most probable switching paths between two metastable states are calculated from the geometric minimum action method (denoted as gMAM paths). It will be interesting to find out whether there is a smooth transition in switching paths when κ increases. We simulate the system through Gillespie's algorithm, and we use principal curve to characterize the averaged switching trajectories (Figure S4). The numerical results suggest that the averaged switching trajectories change smoothly from ODE paths to gMAM paths as κ increases. However, it is a challenging task to prove this argument.

Biological relevance of the parameter values

The biological relevance of the parameter values we use is shown in Table S1. The rates of gene activation/inactivation ($k_G,\ d_G$) are changing over a wide range, with the ratio k_G/d_G being fixed. k_{G0} and k_{R0} should have small values and they are set as 1% and 0.1% of k_G and k_R. After determination of these parameter values, K is calculated to make sure that metastability always exists with κ changing. We find a valid range of the value of K must be from 2754 to 3211, and we set $K = 3000$ for simulations.

Table S1: The biological relevance of the parameter values

Parameters	In Refs.	In Refs. (after unit conversion)	In our paper
k_R (transcription)	0.016 $\sim$ 0.032 mRNAs/s [1]	57.6 $\sim$ 115.2 mRNAs/h	100
k_P (translation)	140 proteins/mRNA/h [4]	140 proteins/mRNA/h	51.5
d_R (mRNA decay)	0.7 /h [3]	0.7 /h	0.7
d_P (protein decay)	1.4 /h [3]	1.4 /h	1.4
d_G (gene inactivation rate)	0.022 /s [2]	79.2 /h	
	0.015 /h [3]	0.015 /h	
k_G/d_G (the ratio of gene activation rate to inactivation rate)	2.8 $\sim$ 20.7 [3]	2.8 $\sim$ 20.7	20

Proof of the argument in the previous section about $O_P = n^k/(n^k + K^k),\ k > 1$

First, let us repeat the deterministic mean-field description of this model:

$$\begin{aligned}\frac{\mathrm{d}m}{\mathrm{d}t} =& \frac{(k_{R0} + k_R \frac{n^k}{n^k+K^k})(k_{G0} + k_G \frac{n^k}{n^k+K^k})}{(d_G + k_{G0} + k_G \frac{n^k}{n^k+K^k})} - d_R m,\\ \frac{\mathrm{d}n}{\mathrm{d}t} =& k_P m - d_P n.\end{aligned} \tag{S16}$$

We define $M(n,k) = n^k/(n^k+K^k) = 1/[1+(K/n)^k]$, and

$$S(n,k) \triangleq \frac{[k_{R1}+M(n,k)][k_{G1}+M(n,k)]}{k_{G2}+M(n,k)} - d_{R1}n \triangleq T[M(n,k)] - d_{R1}n, \tag{S17}$$

where $k_{R1} = k_{R0}/k_R,\ k_{G1} = k_{G0}/k_G,\ k_{G2} = (k_{G0}+d_G)/k_G,\ d_{R1} = d_R d_P/k_R k_P,\ T[M(n,k)] = [k_{R1}+M(n,k)][k_{G1}+M(n,k)]/[k_{G2}+M(n,k)]$. Then solving the equilibrium points of Equation (S16) is equivalent to solving the roots of $S(n,k) = 0$ for each fixed k. If we fix the value of n and let k increase to $+\infty$, $M(n,k)$ will decrease to zero ($n < K$), be equal to 0.5 ($n = K$), or increase to one ($n > K$). Thus, $S_l(n) \triangleq \lim_{k\to+\infty} S(n,k)$ is a piecewise linear function (Figure S5). We compute the derivative

$$\frac{\mathrm{d}T}{\mathrm{d}M} = \frac{[2M+(k_{R1}+k_{G1})](k_{G2}+M)-(k_{R1}+M)(k_{G1}+M)}{(k_{G2}+M)^2} = 1 - \frac{(k_{G2}-k_{G1})(k_{G2}-k_{R1})}{(k_{G2}+M)^2} \tag{S18}$$

According to the definitions we have $k_{G2} > k_{G1}$. If $k_{G2} \geqslant k_{R1}$, $\mathrm{d}T/\mathrm{d}M > 1 - k_{G2}^2/k_{G2}^2 = 0$. If $k_{G2} < k_{R1}$, $\mathrm{d}T/\mathrm{d}M > 1 > 0$. Thus,

$$\frac{\partial S}{\partial k} = \frac{\partial T}{\partial k} = \frac{\mathrm{d}T}{\mathrm{d}M}\frac{\partial M}{\partial k} = \begin{cases} <0, & n<K, \\ >0, & n>K. \end{cases} \tag{S19}$$

And

$$\frac{\partial S}{\partial n} = \frac{\partial T}{\partial n} - d_{R1} = \frac{\mathrm{d}T}{\mathrm{d}M}\frac{\partial M}{\partial n} - d_{R1}, \tag{S20}$$

where

$$\frac{\partial M}{\partial n} = \frac{kK^k/n^{k+1}}{(1+(K/n)^k)^2}. \tag{S21}$$

Here we choose a group of parameters for example: $K_R = 20$, $K_{R0} = 0.2$, $k_P = 49$, $d_R = 0.7$, $d_P = 1.4$, $d_G = 5\times10^4$, $k_G = 10^6$, $k_{G0} = 10^4$, and $K = 300$. In this case, function $S(n,2)$ has three equilibrium points $x_1,\ x_2,\ x_3$ as shown in Figure S5. It is not difficult to verify that $x_1 \approx 1.676$ and $x_3 \approx 852.422$ correspond to stable fixed points of Equation (S16), and $x_2 \approx 130.473$ corresponds to a saddle point. $S(n,2) > 0$ when $n \in [0,x_1) \cup (x_2,x_3)$, and $S(n,2) < 0$ when $n \in (x_1,x_2) \cup (x_3,+\infty)$. Function $S_l(n)$ is a piecewise linear function with two equilibrium points $y_1 = 5/3,\ y_3 \approx 962.358$ and a point of discontinuity $y_2 = 300$. According to Equation (S19) we have

$$S(n,k) = \begin{cases} <0, & n \in [x_1,x_2] \cup [y_3,+\infty],\ k \in \mathbb{N},\ k>2, \\ >0, & n \in [0,y_1] \cup [300,x_3],\ k \in \mathbb{N},\ k>2. \end{cases} \tag{S22}$$

Now we attempt to demonstrate that for all $k > 2$, $S(n,k)$ has three equilibrium points which are located in (y_1,x_1), (x_2,y_2) and (x_3,y_3) separately. When $n \in (y_1,x_1)$,

$$\frac{\partial M}{\partial n} = \frac{k/n}{(K/n)^k+(K/n)^{-k}+2} < \frac{k/y_1}{(300/x_1)^k} < 10^{-3} = d_{R1},\ \forall k>2. \tag{S23}$$

And from the fact that $k_{G2} > k_{R1}$ we know $0 < \mathrm{d}T/\mathrm{d}M < 1$. Hence, $\partial S/\partial n < 0,\ \forall n \in (y_1,x_1),\ k>2$. We obtain that $S(n,k)$ have a unique equilibrium point in (y_1,x_1) as a function of $n\ (k>2)$.

When $n \in (x_2,y_2)$, we define $h(k) = 300/\sqrt[k]{k-3},\ k>3$. Through calculating $\mathrm{d}h/\mathrm{d}k$ we find that $h(k)$ is an increasing function when $k \geqslant 8$, and $245 < h(k) < 300,\ k \geqslant 8$. Then we obtain when $n \in (h(k),y_2)$

$$\frac{\partial M}{\partial n} > \frac{k/y_2}{(300/h(k))^k+1+2} = \frac{1}{300},\ \forall k>8, \tag{S24}$$

$$\frac{\mathrm{d}T}{\mathrm{d}M} > 1 - \frac{(k_{G2}-k_{G1})(k_{G2}-k_{R1})}{k_{G2}^2} = \frac{11}{36},\ \forall k>8. \tag{S25}$$

Thus,

$$\frac{\partial S}{\partial n} > \frac{11}{36\times300} - 10^{-3} > 0,\ \forall k>8. \tag{S26}$$

Since

$$S(h(k),k) = \frac{(\frac{1}{k-2}+0.01)^2}{\frac{1}{k-2}+0.06} - h(k)\cdot10^{-3} < \frac{(\frac{1}{8-2}+0.01)^2}{\frac{1}{8-2}+0.06} - 0.245 < 0,\ \forall k>8, \tag{S27}$$

and $S(y_2,k) = S_l(y_2) > 0,\ \forall k>8$, $S(n,k)$ have a unique equilibrium point as a function of n in the interval $(h(k),y_2)$ (when $k>8$). And in $(x_2,h(k)]$, $S(n,k) < S(n,8) < 0,\ k>8$ (Figure S5). The case of $2 < k \leqslant 8$ can be calculated one by one, and we do not write down the details here.

When $n \in (x_3,y_3)$,

$$\frac{\partial M}{\partial n} = \frac{k/n}{(K/n)^k+(K/n)^{-k}+2} < \frac{k/x_3}{(y_3/300)^k} < 10^{-3} = d_{R1},\ \forall k>2. \tag{S28}$$

And from $0 < \mathrm{d}T/\mathrm{d}M < 1$ we obtain $\partial S/\partial n < 0,\ \forall n \in (x_3,y_3),\ k>2$. Thus, $S(n,k)$ have a unique equilibrium point in (x_3,y_3) as a function of $n\ (k>2)$.

Through computing the eigenvalues of the Jacobian matrix of Equation (S16), we can determine the type of the equilibrium points. This procedure is tedious and we only bring out the results here. The middle equilibrium point is a saddle point, whereas the other two equilibrium points are stable nodes.

References

[1] X. Darzacq, Y. Shav-Tal, V. de Turris, Y. Brody, S. M. Shenoy, R. D. Phair, and R. H. Singer. In vivo dynamics of rna polymerase ii transcription. *Nat. Struct. Mol. Biol.*, 14:796–806, 2007.

[2] J. T. Gerstle and M. G. Fried. Measurement of binding kinetics using the gel electrophoresis mobility shift assay. *Electrophoresis*, 8:725–731, 1993.

[3] L. Mariani, E. G. Schulz, M. H. Lexberg, C. Helmstetter, A. Radbruch, M. Löhning, and T. Höfer. Short-term memory in gene induction reveals the regulatory principle behind stochastic il-4 expression. *Mol. Syst. Biol.*, 6:359, 2010.

[4] B. Schwanhäusser, D. Busse, N. Li, G. Dittmar, J. Schuchhardt, J. Wolf, W. Chen, and M. Selbach. Global quantification of mammalian gene expression control. *Nature*, 473:337–342, 2011.

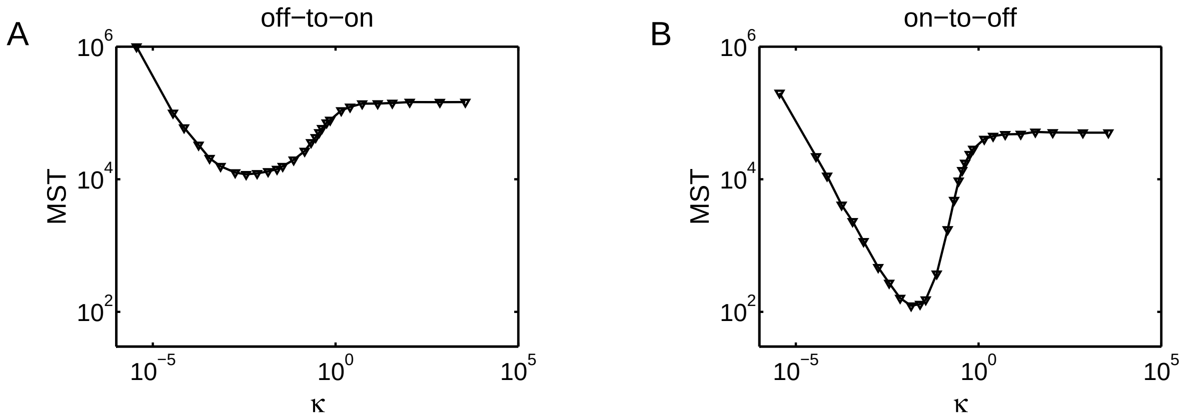

Figure S1: **The mean switching time (MST) as a function of κ when $O_P = n^2/(n^2 + K^2)$.** (A) MST curve of off-to-on switch. (B) MST curve of on-to-off switch. The shapes of MST curves are qualitatively same with the case of $O_P = n/(n+K)$ as shown in Figure 5 of the paper. The parameters are $K_R = 20$, $K_{R0} = 0.2$, $k_P = 51.5$, $d_R = 0.7$, $d_P = 1.4$, $d_G/k_G = 0.05$, $d_G/k_{G0} = 5$, and $K = 300$. We change the value of κ through change the value of d_G. All of the results are obtained from Gillespie's algorithm.

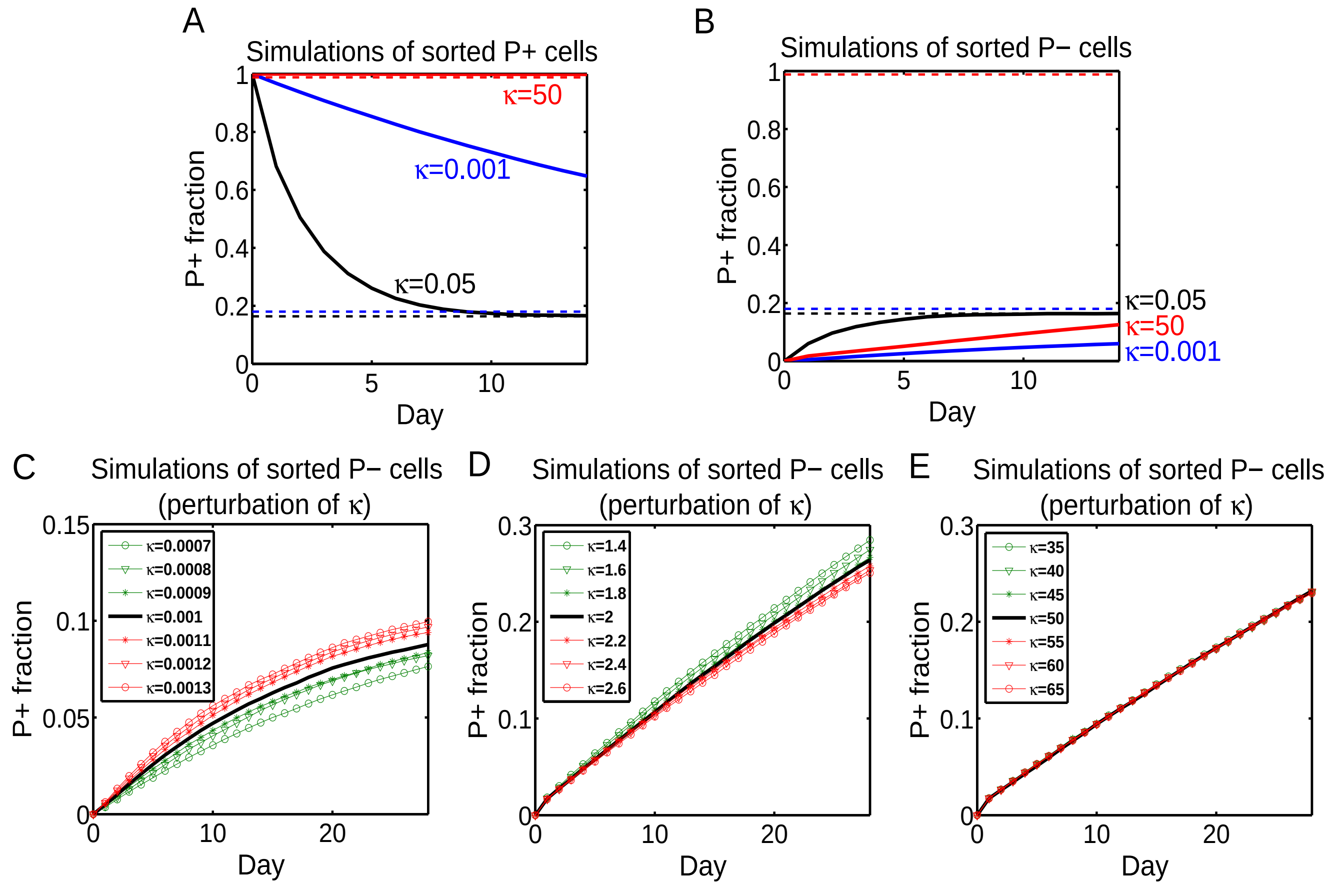

Figure S2: **A method we propose to distinguish the slow, intermediate and fast regimes when** $K = 2754$. (A, B) We simulate the evolution of the two groups for a long time with Gillespie's algorithm. The change of P+ fractions are recorded in solid lines and the final values of P+ fractions are drawn in dashed lines. It is obvious that cells of the intermediate regime have shorter memory, for they rapidly recover to the origin unsorted state (invariant distribution). (C, D, E) If we perturb κ (through changing d_G and keeping the ratios $d_G/k_G,\ d_G/k_{G0}$ and other parameters unchanged), the lines of P+ fractions are shifted in different ways with respect to different regimes. In the slow regime ($\kappa = 0.001$), P+ fraction changes more quickly when κ increases, whereas in the relative fast regime ($\kappa = 2,\ 50$), the time spent for cells to recover to the invariant distribution is longer or unchanged when κ increases. The parameters are $K_R = 100$, $K_{R0} = 0.1$, $k_P = 51.5$, $d_R = 0.7$, $d_P = 1.4$, $d_G/k_G = 0.05$, and $d_G/k_{G0} = 5$.

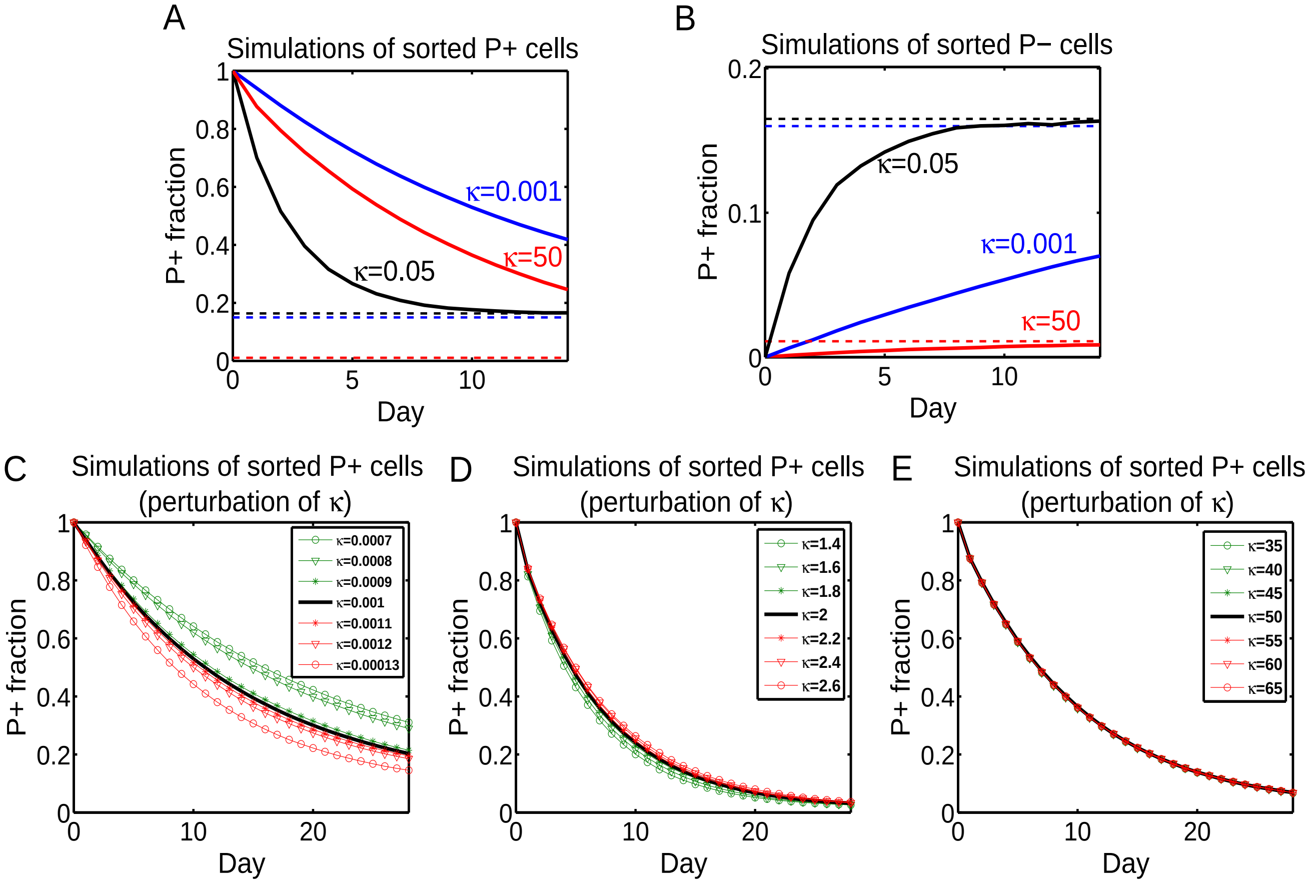

Figure S3: **A method we propose to distinguish the slow, intermediate and fast regimes when $K = 3211$.** (A, B) We simulate the evolution of the two groups for a long time with Gillespie's algorithm. The change of P+ fractions are recorded in solid lines and the final values of P+ fractions are drawn in dashed lines. It is obvious that cells of the intermediate regime have shorter memory, for they rapidly recover to the origin unsorted state (invariant distribution). (C, D, E) If we perturb κ (through changing d_G and keeping the ratios $d_G/k_G,\ d_G/k_{G0}$ and other parameters unchanged), the lines of P+ fractions are shifted in different ways with respect to different regimes. In the slow regime ($\kappa = 0.001$), P+ fraction changes more quickly when κ increases, whereas in the relative fast regime ($\kappa = 2,\ 50$), the time spent for cells to recover to the invariant distribution is longer or unchanged when κ increases. The parameters are the same with Figure S2.

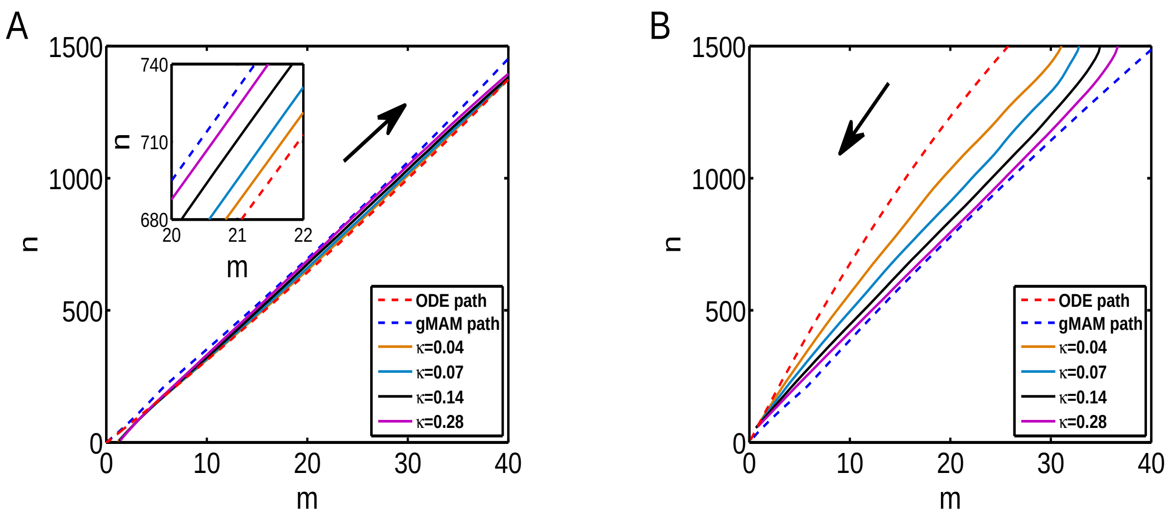

Figure S4: **The change of switching paths as a function of κ.** (A) Off-to-on switching paths. (B) On-to-off switching paths. We use principal curve to characterize the averaged switching trajectories, which change smoothly from ODE paths to gMAM paths as κ increases. The results of $\kappa = 0.04,\ 0.07,\ 0.14,\ 0.28$ are obtained from Gillespie's algorithm. The parameters are the same with Figure S2 except $K = 3000$.

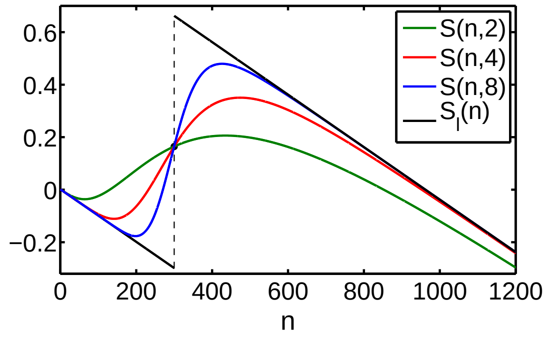

Figure S5: **Illustrations of function** $S(n,k),\ k = 2,4,8$ **and** $S_l(n)$**.** All of the functions pass through the same points when $n = 0$ and $n = 300$. Three equilibrium points of $S(n,2)$ are $x_1 \approx 1.676,\ x_2 \approx 130.473,\ x_3 \approx 852.422$. $S(n,2) > 0$ when $n \in [0, x_1) \cup (x_2, x_3)$, and $S(n,2) < 0$ when $n \in (x_1, x_2) \cup (x_3, +\infty)$. x_1 and x_3 correspond to stable fixed points of Equation (S16), and x_2 corresponds to a saddle point. Similar results can be obtained with respect to $S(n,4)$ and $S(n,8)$. Two equilibrium points of $S_l(n)$ are $y_1 = 5/3,\ y_3 \approx 962.358$. $y_2 = 300$ is a point of discontinuity. y_1 and y_3 both correspond to stable fixed points of Equation (S16). The parameters are $K_R = 20$, $K_{R0} = 0.2$, $k_P = 49$, $d_R = 0.7$, $d_P = 1.4$, $d_G = 5\times10^4$, $k_G = 10^6$, $k_{G0} = 10^4$, and $K = 300$.
